# 

**Published:** 1888-06

**Authors:** 


### CONTENTS OF THIS NUMBER.

PAGE.

**Original Communications—**
Conservatism in Gynæcology. A. R. Jackson ........ 328
Abortion, with Retention of Placenta for five months without Sepsis. E. Wing ........ 334
American Mistletoe as an Oxytocic and Emmenagogue. J. M. G. Carter ........ 336

**Editorial—**
American Medical Association ........ 337
Contamination by Sewage ........ 338

**Society Reports—**
AMERICAN MEDICAL ASSOCIATION—
General Sessions ........ 339–347
Proceedings of Sections ........ 348–365
ASSOCIATION OF AMERICAN MEDICAL EDITORS ........ 365
CONFERENCE OF STATE BOARDS OF HEALTH ........ 366
ILLINOIS STATE MEDICAL SOCIETY ........ 367–376
CHICAGO MEDICAL SOCIETY—
Stated Meeting January 3 ........ 376
Annual Meeting April 2 ........ 379
Stated Meeting April 16 ........ 380
THE GYNÆCOLOGICAL SOCIETY OF CHICAGO—Stated Meeting March 23 ........ 387

**Extracts and Abstracts—**
Special Correspondence of the British Medical Journal—Paris, Vienna, Berlin ........ 390–393
Salicylate of Mercury in Venereal and Cutaneous Diseases ........ 394
Charter of the College of Physicians and Surgeons ........ 395
Transplantation of Nerve from Rabbit to Man ........ 396
Anthrarobin ........ 396
The Avine Origin of Diphtheria ........ 398
Weil's Disease ........ 399
Faith-Cures ........ 399
Extirpation of Constricted Portion of Liver ........ 400
The Parkes Museum of Hygiene ........ 400

**Miscellany** ........ 401–403
**Book Reviews and Notices** ........ 403–407
**Announcements** ........ 408
**List of Advertisers** ........ x
**Publisher's Announcements** ........ x

